# Enhanced Grain Refinement and Precipitation of the IEECAPed Mg-Sm-Zn-Zr Alloy by Nd Addition

**DOI:** 10.3390/ma14195667

**Published:** 2021-09-29

**Authors:** Kun Liu, Sicong Zhao, Changliang Wang, Liping Wang, Yicheng Feng, Dongrong Liu, Jingfang Li, Zhiwei Wang

**Affiliations:** 1 Key Laboratory of Advanced Manufacturing and Intelligent Technology (MOE), School of Material Science and Chemical Engineering, Harbin University of Science and Technology, Harbin 150080, China; lkhust@126.com (K.L.); hlgwangcl@163.com (C.W.); lp_wang2003@126.com (L.W.); fyc7806067@163.com (Y.F.); hitldr@126.com (D.L.); 2 Key Laboratory of Functional Inorganic Material Chemistry (MOE), School of Chemistry and Materials Science, Heilongjiang University, Harbin 150080, China; 3 Aero Engine Corporation of China, Harbin Dongan Engine Co., Ltd., Harbin 150060, China; xztvcn@163.com

**Keywords:** Mg-rare earth alloy, IEECAP, microstructure, mechanical properties

## Abstract

Achieving magnesium-rare earth alloys with excellent mechanical properties remains a challenging goal in the aerospace industry. The integrated extrusion and equal channel angular pressing were employed to refine grain and improve the mechanical properties of Mg-xNd-2.0Sm-0.4Zn-0.4Zr alloys. The effect of Nd element on microstructure and mechanical properties of the extruded and subsequently aged alloys were carried out by varying the amount of the Nd element from 0 wt.% to 2.5 wt.%. The optical microscopy results indicated that the grain size was remarkably refined by the addition of Nd element. The grain size decreased from 29.7 μm to 10.9 μm with increasing of the Nd element from 0 wt.% to 2.5 wt.%. The transmission electron microscopy results showed that the nano-scaled basal lamellar precipitates, prismatic lamellar precipitates and granular precipitates were formed in α-Mg matrix. The amount of the precipitates increased significantly by the addition of Nd. Moreover, the strength of the alloys significantly improved with Nd. Superior strength and considerable plasticity were obtained as the content of Nd element reached 2.0 wt.%, while the tensile strength of the Mg-2.0Nd-Sm-Zn-Zr alloy (315 ± 5 MPa) increased by 35.8% with respect to the Nd-free alloy (232 ± 3 MPa).

## 1. Introduction

Magnesium (Mg) alloys, as the lightest and eco-friendly metal structural material, have received wide attention [1,2,3,4]. Nevertheless, the poor strength of the Mg alloys limits their applications in aerospace industry [5,6]. Among the widely known strategies to improve the strength and plasticity of Mg alloys in industrial applications, severe plastic deformation (SPD) processing is an ideal choice. This is especially seen in equal channel angular pressing (ECAP), which induces enormous levels of strain into the alloys without changing the shape of the sections of the sample [7,8,9,10]. However, ECAP is mainly focused on Mg-Al alloys [11,12]. The mechanical properties of ECAPed Mg-Al alloys could not meet the further application of aerospace now. In this regard, more researchers have been paying attention to Mg-rare earths (RE) alloys. Extensive investigations have proved that RE could improve the mechanical properties of Mg alloys significantly. Nevertheless, the study for ECAPed Mg-light RE alloys is insufficient. Recently, samarium (Sm) has been widely used to improve the strength of Mg alloys. The cost performance and precipitation strengthening ability of Sm-containing Mg alloys are excellent [13,14,15,16]. Moreover, the neodymium (Nd) element is a high-efficiency light RE element used to enhance the mechanical properties of Mg alloys. The addition of Nd element in Mg-Y-Zr alloys could improve its strength due to precipitation strengthening and grain refinement, according to the studies by Xu et al. [17]. Wang et al. [18] showed that the Nd could endow the Mg-2Zn-0.46Y alloy with homogeneous and fine grain structures. Guadalupe et al. [19] applied the Nd addition to reduce the yield asymmetry of Mg-Zn alloys. In general, the type of the precipitates consists of β series precipitates and *γ* series precipitates in Mg-Nd(-Zn) alloys [20].

For these reasons, Mg-Nd-Sm-Zn-Zr alloys were designed and prepared according on the advantages of multi-component alloying. In the present work, in order to simplify the traditional ECAP techniques, multi-pass of ECAP process are optimized to one pass. Moreover, in order to maintain a considerable strain levels, the angle *ϕ* between the equal channels is reduced to 90^°^. The integrated extrusion and equal channel angular pressing (IEECAP) could significantly improve extrusion efficiency, and the size of extruded bar are not limited by the extrusion mold [21]. Therefore, the IEECAP was employed to refine grain and enhance the mechanical properties of the Mg-Nd-Sm-Zn-Zr alloys. Furthermore, the aging treatment is an extremely effective way to enhance the strength of the Mg-RE alloys. The Nd element played a key role in the microstructure and mechanical properties of the IEECAPed and subsequently aged Mg-Nd-Sm-Zn-Zr alloys. However, the effect of Nd on microstructure and mechanical properties of Mg-Nd-Sm-Zn-Zr alloys is studied inadequately.

Herein, the IEECAP and subsequent ageing were employed to enhance the mechanical properties of the Mg-Nd-Sm-Zn-Zr alloy. The effect of Nd element on grain refinement and precipitation of the Mg-Nd-Sm-Zn-Zr alloy was carried out by varying the amount of the Nd element from 0 wt.% to 2.5 wt.%.

## 2. Materials and Methods

**Figure 1:**
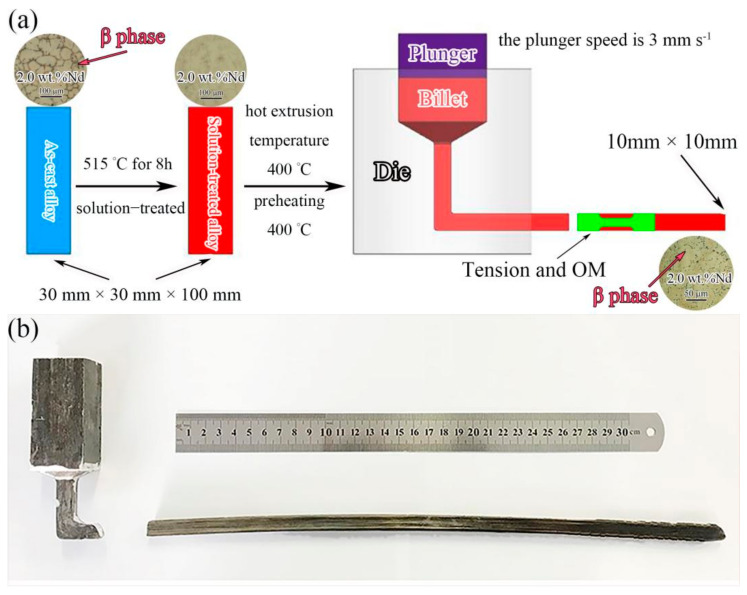
IEECAP process (**a**) and macrograph of the IEECAPed sample (**b**).

The compositions of Mg-xNd-2.0Sm-0.4Zn-0.4Zr alloys (x = 0 wt.%, 0.5 wt.%, 1.0 wt.%, 1.5 wt.%, 2.0 wt.% and 2.5 wt.%) alloys were shown in Table 1. The alloys were prepared from Mg ingots (Mg > 99.9 wt.%), Zn ingots (Zn > 99.99 wt.%), Mg-30 wt.% Sm alloy, Mg-30 wt.% Zr alloy and Mg-25 wt.% Nd alloy. The alloys were melted at 780 °C under the protective atmosphere of CO_2_ and SF_6_. The melt was stirred for 300 s, then standing for 180 s. The alloys were then cast into metal mold at 720 °C. The material of the metal mold was low-carbon steel. In order to prevent gas holes and cold shut of the ingots, the metal mold was preheated at 200 °C for more than 5 h prior to casting. The schematic diagram of the IEECAP process and macrograph of the IEECAPed sample were shown in Figure 1. The range of the extrusion pressure of the IEECAP is 700 MPa to 1100 MPa and the speed of the plunger is approximately 3 mm s^−1^. The IEECAPed samples were aged at 190 °C for 18 h. The mechanical properties were characterized by tensile tests. The tensile test fracture morphologies of the alloys were observed and analyzed by scanning electron microscope (SEM, Apreo C, Thermo Fisher Scientifific Inc., Hillsboro, OR, USA). The microstructure was observed and analyzed by optical microscopy (OM, OLYMPUS-GX71, Olympus Co., Tokyo, Japan) and transmission electron microscopy (TEM, JEM-2100, JEOL Co. Ltd., Tokyo, Japan). In this work, the linear intercept method was employed to quantitatively analyze the average grain size.

## 3. Results and Discussion

### 3.1. Microstructure

**Figure 2:**
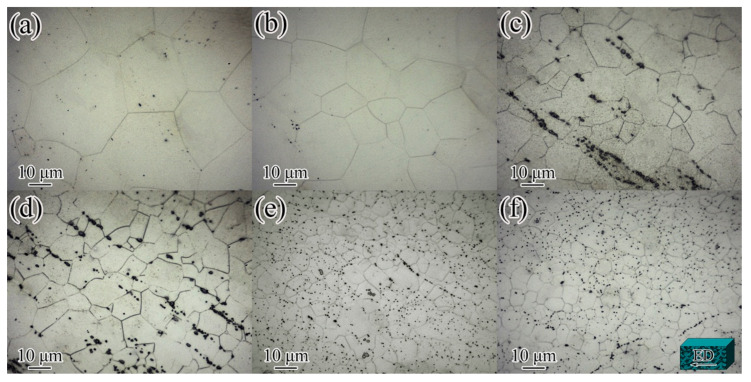
Optical microstructure of the IEECAPed and subsequently aged Mg-xNd-2.0Sm-0.4Zn-0.4Zr alloys with different Nd content (wt.%): (**a**) 0, (**b**) 0.5, (**c**) 1.0, (**d**) 1.5, (**e**) 2.0 and (**f**) 2.5.

**Figure 3:**
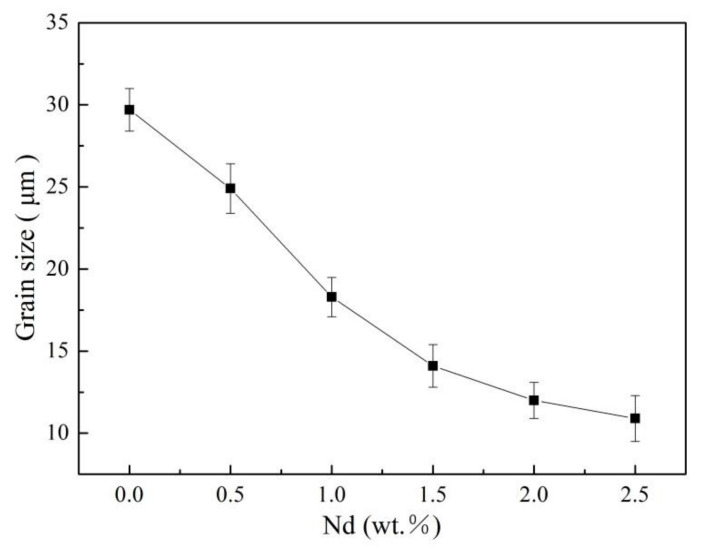
Grain size of the IEECAPed and subsequently aged Mg-xNd-2.0Sm-0.4Zn-0.4Zr alloys by Nd addition.

The initial microstructure of the Mg-2.0Nd-2.0Sm-0.4Zn-0.4Zr alloy before the IEECAP can be observed in Figure 1. The microstructure of the as-cast Mg-2.0Nd-2.0Sm-0.4Zn-0.4Zr alloy consisted of equiaxed grains and the β phase [3]. After solution treatment, β phase was dissolved in α-Mg matrix. The microstructure of IEECAPed and subsequently aged Mg-xNd-2.0Sm-0.4Zn-0.4Zr alloys with different content of Nd element were investigated by OM. As shown in Figure 2, the microstructure of the alloys mainly consisted of equiaxed grains, and the addition of Nd element could remarkably refine the grain size. In order to quantitatively analyze the grain size, the relation curve of grain size with Nd addition was given in Figure 3. In the Nd-free alloy, it exhibited coarse grain (29.7 μm). When Nd element increased to 2.5 wt.%, the grain size was the finest (10.9 μm). It is worth mentioning that some second phases formed directionally in the alloys when the Nd element exceed 1.0 wt.%. However, directionality of distribution of the second phases was not obvious as the Nd element exceed 2.0 wt.%. According to the morphology of the second phases and the composition of the alloys, the second phases is β phase [22]. In general, the grain refinement is attributed to the solute drag mechanism and second phase pinning mechanism. During the extrusion process, more dislocations can be pinned by the obstacles (solute atoms and second phase particles, etc.), which leads to an increase in the density of dislocations, and the nucleation of recrystallized grain can be further promoted [23]. Moreover, the β particles can block grain boundaries and prevent recrystallized grain growth [23].

**Figure 4:**
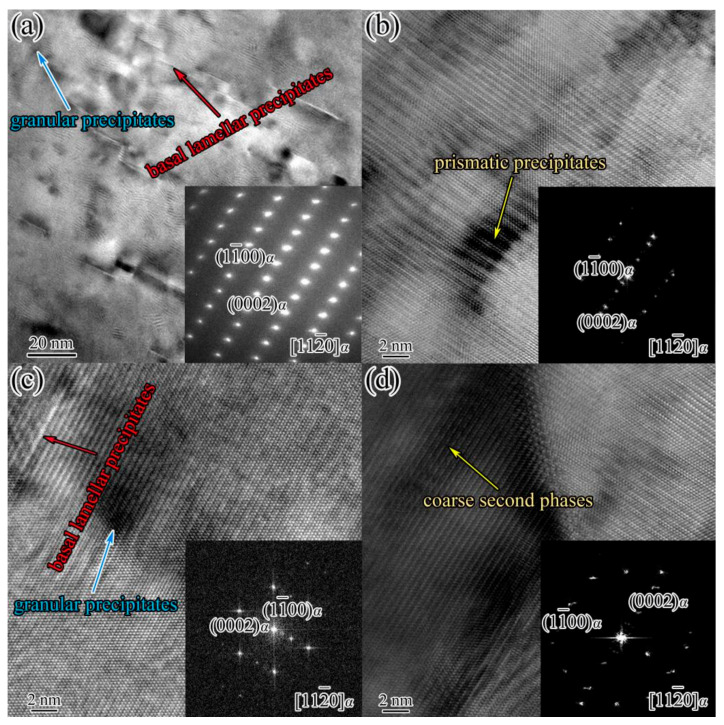
The TEM micrographs and SAED patterns of the IEECAPed and subsequently aged Mg-2.0Sm-0.4Zn-0.4Zr alloy along [11$\bar{2}$0]_α_ zone axis. (**a**) TEM micrograph and SAED patterns of the basal lamellar precipitates and granular precipitates; (**b**) HRTEM image of the prismatic lamellar precipitates; (**c**) HRTEM image of the granular precipitates and basal lamellar precipitates; (**d**) HRTEM image of the coarse second phases.

The TEM observation and selected area electron diffraction (SAED) patterns was employed to investigate the nano-scaled precipitates in the IEECAPed and subsequently aged Mg-2.0Sm-0.4Zn-0.4Zr alloy. Figure 4a showed the TEM micrographs of the alloys along [11$\bar{2}$0]_α_ zone axis. It can be seen that the nano-scaled lamellar precipitates and granular precipitates occurred in α-Mg matrix. Moreover, most of the lamellar precipitates formed in the basal plane of α-Mg matrix, a few lamellar precipitates formed in the prismatic planes. In addition, fewer coarse second phases existed in α-Mg matrix. To further study the nano-scaled prismatic lamellar precipitates, basal lamellar precipitates, granular precipitates and coarse second phases, the high-resolution transmission electron micrography (HRTEM) and fast Fourier transform (FFT) were employed. The results are shown in Figure 4b–d, respectively. The nano-scaled prismatic lamellar precipitates, basal lamellar precipitates and granular precipitates were completely coherent with α-Mg matrix. The granular precipitates were typically less than 5 nm. The length of the lamellar precipitates was approximately 20 nm. According to the morphology, size and the orientation relationship, the basal lamellar precipitates, granular precipitates, prismatic lamellar precipitates and coarse second phases are identified as *γ*′ phase, cluster of atoms, β′ phase and β_1_ phase, respectively [20].

**Figure 5:**
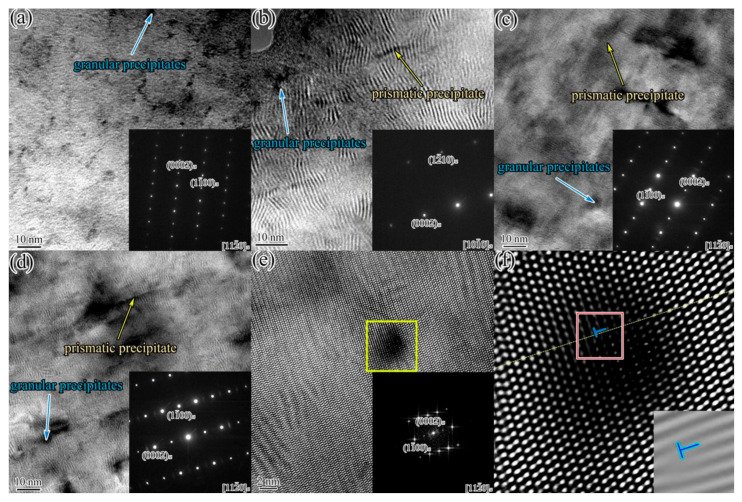
TEM micrographs and SAED patterns of the IEECAPed and subsequently aged Mg-xNd-2.0Sm-0.4Zn-0.4Zr alloys with different Nd content (wt.%): (**a**) 0.5, (**b**) 1.0, (**c**) 1.5, (**d**) 2.0. (**e**) HRTEM images of the granular precipitates in Mg-2.0Nd-2.0Sm-0.4Zn-0.4Zr along [11$\bar{2}$0]_α_ zone axis; (**f**) IFFT image and the lattice fringes obtained by IFFT of the selected area from (**e**).

High magnification TEM micrographs of the IEECAPed and subsequently aged Mg-xNd-2.0Sm-0.4Zn-0.4Zr alloys (x = 0.5 wt.%, 1.0 wt.%, 1.5 wt.% and 2.0 wt.%) were employed to study the effect of the Nd element of precipitates. As shown in Figure 5a–d, the prismatic lamellar precipitates and the granular precipitates still existed in α-Mg matrix. Moreover, the size of these precipitates showed no visible changes compared with the Nd-free alloy. For further research of the granular precipitates in the alloys, the HRTEM and FFT image of the granular precipitates in the Mg-2.0Nd-2.0Sm-0.4Zn-0.4Zr alloy along [11$\bar{2}$0]_α_ zone axis were shown in Figure 5e, which showed that the granular precipitates were still completely coherent with the α-Mg matrix. The inverse Fourier-filtered transformation (IFFT) was employed to further analyze the effect of the granular precipitates on the atomic arrangement in α-Mg matrix. Figure 5f shows the IFFT image and the lattice fringes obtained by IFFT corresponding to the box in Figure 5e. As shown in Figure 5f, the interatomic distance around granular precipitates slightly differ in α-Mg matrix, which was attributed to the lattice distortions caused by the solute atoms. More importantly, these non-negligible local distortions formed within the granular precipitates, and they were conducive to improve the mechanical properties [24].

**Figure 6:**
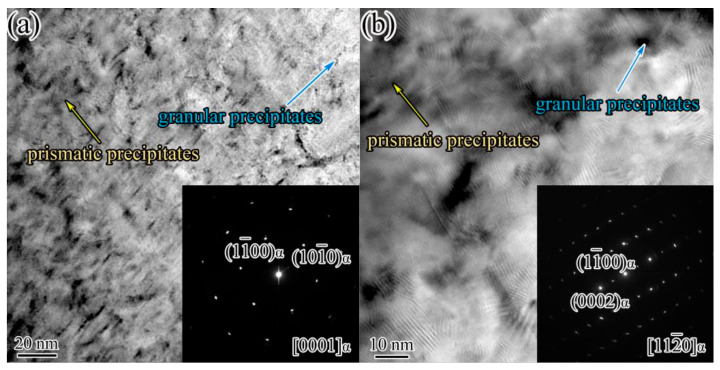
The TEM micrographs and SAED patterns of the IEECAPed and subsequently aged Mg-2.5Nd-2.0Sm-0.4Zn-0.4Zr. (**a**) TEM micrograph and SAED patterns along [0001]_α_ zone axis of the alloy; (**b**) TEM micrograph and SAED patterns along [11$\bar{2}$0]_α_ zone axis of the alloy.

The TEM micrograph of the IEECAPed and subsequently aged Mg-2.5Nd-2.0Sm-0.4Zn-0.4Zr alloys along [0001]_α_ zone axis and [11$\bar{2}$0]_α_ zone axis are shown in Figure 6a,b, respectively. Prismatic lamellar precipitates and granular precipitates existed in aged Mg-2.5Nd-2.0Sm-0.4Zn-0.4Zr alloy. The amount of the precipitates significantly increased than the Nd-free alloy. In addition, the morphology of the granular precipitates on [0001]_α_ did not evidently change compare to the [11$\bar{2}$0]_α_. Therefore, the addition of the Nd element could observably promote the formation of the precipitates, especially for the granular precipitates and prismatic lamellar precipitates.

### 3.2. Mechanical Properties and Fracture Analysis

**Figure 7:**
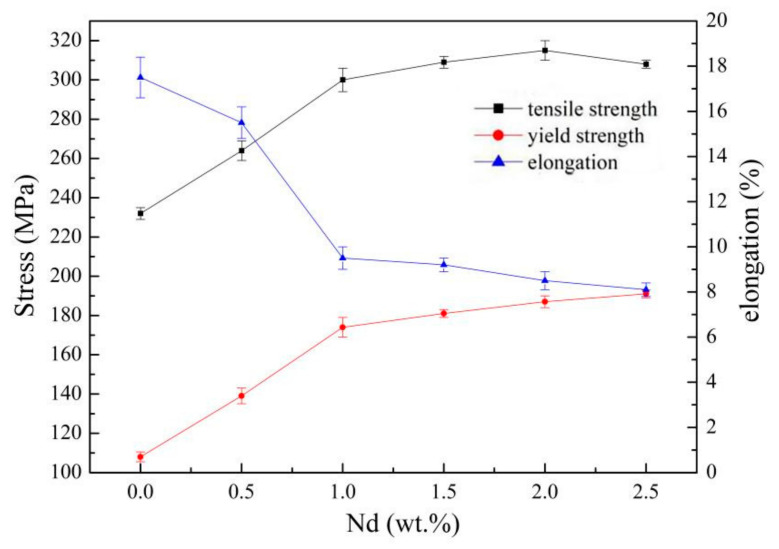
Tensile properties and elongation of the IEECAPed and subsequently aged Mg-xNd-2.0Sm-0.4Zn-0.4Zr alloys.

The change curve of tensile property of the IEECAPed and subsequently aged Mg-xNd-2.0Sm-0.4Zn-0.4Zr alloys as the function of the content of Nd element were shown in Figure 7. Upon increasing the Nd from 0 wt.% to 2.0 wt.%, tensile strength of the alloys increased from 232 ± 3 MPa to 315 ± 5 MPa, the yield strength of the alloys increased from 108 ± 2.5 MPa to 187 ± 3 MPa, and the elongation decreased from 17.5 ± 0.9% to 8.5 ± 0.4%. There are two main reasons for the improvement of the strength of the alloys. First, according to the Hall-Petch formula, grain refinement significantly improved the strength. The grain boundary area was increased by the grain refinement, and the grain boundary acts as a strong barrier to the slip transmission. Second, the precipitates increased obviously in the alloys because of the increase of the Nd element, which hinders the dislocation movement during the tension process.

**Figure 8:**
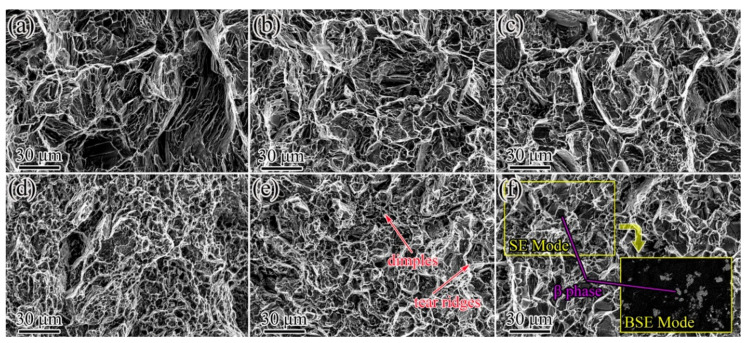
Tensile test of fracture microstructure of the IEECAPed and subsequently aged Mg-xNd-2.0Sm-0.4Zn-0.4Zr alloys with different Nd content (wt.%): (**a**) 0, (**b**) 0.5, (**c**) 1.0, (**d**) 1.5, (**e**) 2.0 and (**f**) 2.5.

Figure 8 showed the fracture surfaces of the IEECAPed and subsequently aged Mg-xNd-2.0Sm-0.4Zn-0.4Zr alloys. It showed that the fractographs of the alloys mainly consisted of tear ridges. In addition to that, some dimples existed in the alloys as the content of Nd exceed 1.0 wt.%, and the fractographs were progressively refined by addition of the Nd element. The fractographs of the alloys can be classified as transgranular fracture. Figure 8f showed the fracture surfaces combined with secondary electron (SE) and back scattering electron (BSE) modes for Mg-2.5Nd-2.0Sm-0.4Zn-0.4Zr alloys. The β phase is a hard and brittle phase, which act as the source of cracks and induce fracture.

## 4. Conclusions

1. The Nd element can remarkably refined the grain size of the IEECAPed and subsequently aged Mg-xNd-2.0Sm-0.4Zn-0.4Zr alloys. The grain size of the alloys decreased from 29.7 μm to 10.9 μm with the content of Nd element from 0 wt.% to 2.5 wt.%.
2. The nano-scaled basal lamellar precipitates, prismatic lamellar precipitates, granular precipitates and coarse second phases occurred in IEECAPed and subsequently aged Mg-xNd-2.0Sm-0.4Zn-0.4Zr alloys. The amount of the precipitates increased significantly with the addition of the Nd element, especially with respect to the granular precipitates and prismatic lamellar precipitates.
3. The tensile property of the alloys significantly improved by the addition of Nd. Superior strength and considerable plasticity were observed, as the content of Nd element reaching to 2.0 wt.%, the tensile strength of the Mg-2.0Nd-Sm-Zn-Zr alloy (315 ± 5 MPa) increased by 35.8% with respect to the Nd-free alloy (232 ± 3 MPa). The fractograph of the alloys were classified as transgranular fracture.

## Figures and Tables

**Table 1:** Nominal compositions and actual compositions of Mg-xNd-2.0Sm-0.4Zn-0.4Zr alloys.

Nominal Compositions (wt.%)	Actual Compositions (wt.%)				
	Nd	Sm	Zn	Zr	Mg
Mg-2.0Sm-0.4Zn-0.4Zr	-	2.03	0.41	0.42	Bal.
Mg-0.5Nd-2.0Sm-0.4Zn-0.4Zr	0.51	2.05	0.42	0.41	Bal.
Mg-1.0Nd-2.0Sm-0.4Zn-0.4Zr	1.04	2.01	0.41	0.42	Bal.
Mg-1.5Nd-2.0Sm-0.4Zn-0.4Zr	1.54	2.07	0.40	0.42	Bal.
Mg-2.0Nd-2.0Sm-0.4Zn-0.4Zr	2.03	2.05	0.41	0.41	Bal.
Mg-2.5Nd-2.0Sm-0.4Zn-0.4Zr	2.51	2.04	0.42	0.41	Bal.

## Data Availability

The data presented in this study are available on request from the corresponding author.
